# Incidence of Adult Acquired Flatfoot Deformity Referred to Specialist Care in Sweden

**DOI:** 10.1002/jfa2.70042

**Published:** 2025-02-27

**Authors:** Ida Osbeck, Maria Cöster, Isam Atroshi

**Affiliations:** ^1^ Department of Clinical Sciences Lund–Orthopedics Lund University Lund Sweden; ^2^ Department of Clinical Sciences Malmö ‐ Orthopedics Lund University Lund Sweden; ^3^ Department of Surgical Sciences Uppsala University Uppsala Sweden; ^4^ Department of Orthopedics Hässleholm Hospital Hässleholm Sweden

**Keywords:** AAFD, incidence, register study

## Abstract

**Introduction:**

Adult acquired flatfoot deformity (AAFD) is a disabling condition that may require complex surgical treatment. Little is known about the incidence of AAFD in the general population and specifically of AAFD requiring specialist care. We aimed to describe the incidence of AAFD referred to specialist care in the Swedish general population.

**Methods:**

We conducted a nation‐wide epidemiological register study to estimate the incidence of referred AAFD in the general population. We retrieved data from the Swedish National Patient Register. All individuals aged 16 years or older, with a first‐time diagnosis of AAFD (ICD‐10 code M214) between 2007 and 2018 were identified. Total incidences, change over time, and gender‐specific and age‐specific incidences per 100,000 person‐years were calculated using population size data from Statistics Sweden. Incidences were compared using the Poisson test.

**Results:**

The incidence rate of referred AAFD in the general population was 23.0 (95% CI 22.7–23.3) per 100,000 person‐years. The incidence rate in women was 30.4 (95% CI 29.9–30.8) and in men was 15.4 (95% CI 15.1–15.8). The highest incidence rates were found in the age Group 61–75 years. The incidence rates varied significantly across the 21 regions in Sweden. The age‐standardized and sex‐standardized incidence rates ranged from 8.3 (95% CI 7.2–9.4) to 69.1 (95% CI 62.4–75.8).

**Conclusion:**

AAFD requiring referral to specialist care is common in the general population. Women had nearly twice the incidence of AAFD compared to men. Large unexplained regional variations in the incidence rates exist.

**Abbreviations**
AAFD: adult acquired flatfoot deformity
NPR: the Swedish National Patient Register

## 1 Introduction

**FIGURE 1:**
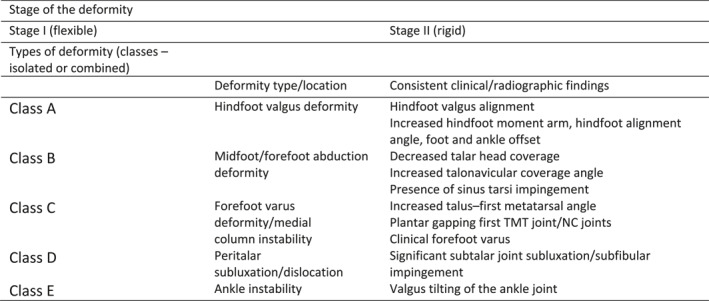
Consensus Group classification of adult acquired flatfoot deformity*. *Adapted from Myerson et al. NC, naviculocuneiform; TMT, tarsometatarsal.

Adult acquired flatfoot deformity (AAFD) is a disabling condition that may require complex surgical treatment to correct the deformity [1, 2]. The condition varies in severity from mild symptoms without deformity to severe deformity (Figure 1). Although the exact cause of AAFD is not known, posterior tibial tendon dysfunction is believed to be part of the pathology and the term has been used to refer to AAFD [1].

There is scarcity of data regarding the incidence and prevalence of AAFD in the general population. Using a combination of survey and clinical assessment, a study from England estimated the prevalence of flatfoot “pathology” in women above 40 years of age, registered at a county general practice, at 6.6% and the prevalence of symptomatic flatfoot at 3.3% [2]. A study using a podoscope to diagnose AAFD in a sample of about 1000 individuals randomly selected from the population of a Spanish municipality with a population of approximately 24,000, estimated the prevalence in the age Group 40 years or older at 19% [3]. A study from the United States involving 185 acute care and ambulatory surgery centers in South Carolina found that the incidence of corrective surgery for AAFD per 100,000 increased from 0.26 in 1996 to 3.04 in 2014 [4]. These relatively limited data about the prevalence and incidence of AAFD in regions in Europe and North America highlight the need for studies in other regions, and the healthcare register in Sweden is a valuable nation‐wide data source.

Although the prevalence in women is approximately two to three fold that in men, and the reported peak incidence is in the age Group 50–60 years, men and persons younger than 40 years account for a substantial proportion of patients with AAFD [5, 6, 7]. A recent scoping review highlighted the limited knowledge about AAFD‐related aspects, such as the prevalence and incidence of posterior tibial tendon dysfunction in most ages, in different regions, and in males. Thus, there are gaps in currently available data regarding different demographics, regions, and progression of the condition. The extent to which individuals with AAFD in the general population experience symptoms severe enough to justify referral to specialist care is unknown. It is also unknown whether the incidence of referred AAFD varies in different regions.

Knowledge about the incidence rate of a disease is important in public health planning, including resource allocation and planning of interventions and prevention programs as well as in early diagnosis and treatment impacting quality of life. This information is also important when making decisions regarding research funding. By describing the incidence rates in different subgroups and the incidence rate over time, trends can be identified and analyzed in relation to other disorders and public health measures. This could guide further research investigating potential underlying risk factors and protective factors, contributing to a deeper understanding of disease development. Determining incidence rates across different regions can reveal geographic variations that may suggest differences in the prevalence of underlying risk factors or in access to and quality of healthcare as well as access to foot surgeons, warranting further investigation.

The aims of this study were to estimate the incidence of AAFD referred to specialist care in the Swedish general population, to analyze changes in incidence over time, and to explore possible regional variations. We hypothesized that the incidence of referred AAFD had increased due to improved understanding and recognition of AAFD.

## 2 Materials and Methods

This was a population‐based study using data from a nation‐wide register in Sweden. The study was approved by the Swedish Ethical Review Authority (2020‐11‐11, ref. 2020–05550 and 2021–02056).

### 2.1 The Swedish National Patient Register

The Swedish National Board of Health and Welfare manage several registers including the National Patient Register (NPR). Reporting to the NPR is mandatory for all county councils. Since 1987, the NPR includes all inpatient care in Sweden. All outpatient doctor visits including day surgery are included in the NPR since 2001. For patients referred to specialist care where the referral is either rejected or does not result in a clinical visit, no diagnosis code is registered in the NPR. Information is delivered from the 21 county councils and private healthcare providers to the National Board of Health and Welfare every month. The coverage has been very high for inpatient care, and although it was initially lower for outpatient care, it improved gradually to a high level. Frequent quality and validity controls of the NPR are made, and if data inaccuracy is suspected, new data can be requested. The NPR contains data including personal registration number (unique for every resident in Sweden), healthcare provider, county, diagnoses (primary diagnosis and up to 19 secondary diagnoses), and date of healthcare visits [8]. The Swedish version of the International Statistical Classification of Diseases and Related Health Problems (ICD‐10‐SE) is used for the coding of diagnoses.

### 2.2 Data Extraction

We applied for data extraction to the Swedish National Board of Health and Welfare in November 2021 for all patient visits to a medical doctor in secondary and tertiary healthcare facilities from January 1, 2007, to December 31, 2018, in which the ICD‐10‐SE code M214 had been registered. The study period of 2007–2018 was selected to maximize the number of years included while ensuring that the dataset could be evenly divided into two time periods for a comparative analysis of incidence trends over time. In addition, years affected by the COVID‐19 pandemic were excluded, as they did not accurately reflect referral patterns or clinical visits to orthopedic centers in Sweden. We also requested data regarding patient characteristics (coded ID‐number, sex, and age), date of the visit, the visited healthcare facility, and the region of the country.

We received the data from the Swedish National Board of Health and Welfare in September 2022.

### 2.3 Inclusion Criteria

The inclusion criteria were patients aged 16 years or older with a first‐time diagnosis of AAFD made by a medical doctor at a secondary or tertiary healthcare facility. The diagnostic code used exclusively for AAFD is M214 described in the ICD‐10 as “Flat foot [pes planus] (acquired)”.

### 2.4 Statistical Analyses

We identified all individuals with a first‐time diagnosis of AAFD. Annual data on the population size were retrieved from Statistics Sweden [9]. For each year, the population data on December 31st, of that year, was used. We calculated the incidence rate as number of cases per 100,000 person‐years with 95% confidence intervals (CIs). We analyzed changes in the incidence rate over time (years 2007–2012 and 2013–2018) and compared the incidence rates with the Poisson test. County, age, and gender adjusted incidence rates were also calculated. The at‐risk population was standardized with weights from the Swedish general population using data from Statistics Sweden [9]. Statistical significance was defined as a *p*‐value < 0.05.

For statistical analyses, we used IBM SPSS Statistics 28 Windows (IBM, Corporation, Armonk, NY, USA) and *R* Core Team (2022). R: A language and environment for statistical computing. *R* Foundation for Statistical Computing, Vienna, Austria. URL https://www.R‐project.org/. Version 4.2.2.

## 3 Results

During the 12‐year study period (2007–2018), a diagnosis of AAFD (ICD‐10 code M214) was registered at 54,965 patient visits by a medical doctor at a secondary or tertiary healthcare facility. A total of 21,766 patients (14,547 [66.8%] women) aged 16 years or older received a first‐time diagnosis of AAFD during the study period. In 21,127 (97.1%) first‐time visits, M214 was the main diagnosis. The incidence rate (95% CI) per 100,000 person‐years was 23.0 (22.7–23.3) (Table 1). The incidence rate increased significantly from 19.7 (19.3–20.1) during 2007–2012 to 26.2 (25.7–26.6) during 2013–2018 and the incidence rate ratio was 1.33 (95% CI 1.30–1.37).

**TABLE 1:** Incidence of referred adult acquired flatfoot deformity (diagnosis code M214) per 100,000 person‐years in the Swedish general population during the years 2007–2018.

	2007–2018	2007–2012	2013–2018
Total	23.0 (22.7–23.3)	19.7 (19.3–20.1)	26.2 (25.7–26.6)
Women	Incidence (95% CI)	Incidence (95% CI)	Incidence (95% CI)
Unadjusted	30.5 (30.0–31.0)	26.6 (25.9–27.3)	34.3 (33.6–35.1)
Age‐adjusted	30.4 (29.9–30.8)	26.5 (25.8–27.1)	34.1 (33.4–34.8)
Age group			
16–30	15.9 (15.1–16.6)	14.5 (13.5–15.5)	17.2 (16.1–18.2)
31–45	16.6 (15.8–17.4)	14.7 (13.7–15.7)	18.5 (17.3–19.6)
46–60	41.2 (40.0–42.4)	35.6 (34.0–37.2)	46.5 (44.7–48.3)
61–75	51.8 (50.4–53.3)	46.1 (44.1–48.1)	57.0 (54.9–59.1)
76+	30.2 (28.7–31.6)	24.5 (22.7–26.4)	35.7 (33.4–37.9)
Men			
Unadjusted	15.4 (15.0–15.7)	12.6 (12.1–13.1)	18.0 (17.5–18.5)
Age‐adjusted	15.4 (15.1–15.8)	12.6 (12.2–13.1)	18.1 (17.5–18.6)
Age group			
16–30	12.9 (12.2–13.5)	10.5 (9.7–11.4)	15.1 (14.1–16.1)
31–45	10.4 (9.8–10.9)	8.4 (7.6–9.1)	12.3 (11.4–13.2)
46–60	17.2 (16.5–18.0)	14.9 (13.9–16.0)	19.4 (18.3–20.5)
61–75	22.5 (21.5–23.5)	18.0 (16.7–19.2)	26.6 (25.1–28.0)
76+	15.6 (14.3–16.9)	12.7 (11.0–14.4)	18.2 (16.3–20.1)

The incidence rate was consistently higher in women than in men (Table 1). The incidence rate was 30.4 (29.9–30.8) in women and 15.4 (15.1–15.8) in men and the incidence rate ratio was 1.99 (95% CI 1.93–2.04). In both women and men, the incidence increased above age 45. The highest incidence rates were observed in the age Group 61–75 years with incidence rates of 51.8 (50.4–53.3) in women and 22.5 (21.5–23.5) in men.

### 3.1 Regional Variations

The incidence rate (95% CI) varied from 8.3 (7.2–9.4) in Västerbotten and 8.6 (7.5–9.6) in Värmland to 38.7 (37.8–39.5) in Stockholm and 69.1 (62.4–75.8) in Gotland. The increase in the incidence rate from 2007–2012 to 2013–2018 was significant in 9 of 21 Swedish regions, but in 4 regions, there was a statistically significant decrease (Table 2).

**TABLE 2:** Incidence of referred adult acquired flatfoot deformity (diagnosis code M214) per 100,000 person‐years in the Swedish general population and in the 21 regions of Sweden during the years 2007–2018.

	2007–2018		2007–2012		2013–2018		2013–2018 versus 2007–2012	
	Incidence (95% CI)		Incidence (95% CI)		Incidence (95% CI)		Unadjusted	
	Crude	Adjusted^a^	Crude	Adjusted^a^	Crude	Adjusted^a^	Incidence rate ratio (95% CI)	*p* ^b^
Total	23.0 (22.7–23.3)	—	19.7 (19.3–20.1)	—	26.2 (25.7–26.6)	—	1.33 (1.30–1.37)	< 0.001
Region								
Stockholm	36.9 (36.1–37.7)	38.7 (37.8–39.5)	25.3 (24.3–26.3)	26.5 (25.5–27.6)	47.5 (46.2–48.8)	49.8 (48.4–51.2)	1.88 (1.79–1.97)	< 0.001
Uppsala	15.3 (14.0–16.6)	15.7 (14.4–17.1)	15.6 (13.7–17.6)	16.1 (14.1–18.0)	14.9 (13.1–16.8)	15.4 (13.5–17.3)	0.96 (0.80–1.14)	0.629
Södermanland	12.2 (10.9–13.5)	11.8 (10.6–13.1)	13.8 (11.8–15.8)	13.4 (11.5–15.3)	10.8 (9.1–12.5)	10.4 (8.7–12.0)	0.78 (0.63–0.98)	0.028
Östergötland	12.1 (11.1–13.1)	12.1 (11.1–13.1)	11.1 (9.7–12.5)	11.0 (9.6–12.5)	13.1 (11.6–14.6)	13.1 (11.6–14.6)	1.18 (0.99–1.40)	0.066
Jönköping	15.2 (13.8–16.5)	15.2 (13.9–16.5)	13.0 (11.3–14.8)	13.1 (11.4–14.9)	17.2 (15.2–19.2)	17.2 (15.2–19.2)	1.32 (1.10–1.58)	0.0022
Kronoberg	27.9 (25.5–30.3)	28.0 (25.5–30.4)	32.9 (29.1–36.6)	33.0 (29.3–36.8)	23.1 (20.0–26.2)	23.1 (20.0–26.1)	0.70 (0.59–0.84)	< 0.001
Kalmar	16.6 (15.0–18.2)	15.9 (14.4–17.5)	20.4 (17.8–23.0)	19.7 (17.2–22.2)	12.8 (10.8–14.9)	12.2 (10.3–14.2)	0.63 (0.51–0.77)	< 0.001
Gotland	71.6 (64.7–78.5)	69.1 (62.4–75.8)	71.1 (61.4–80.9)	69.7 (60.1–79.3)	72.2 (62.4–82.0)	68.5 (59.2–77.9)	1.01 (0.83–1.24)	0.883
Blekinge	17.6 (15.5–19.7)	17.0 (14.9–19.0)	17.6 (14.6–20.5)	17.3 (14.3–20.2)	17.6 (14.6–20.5)	16.6 (13.8–19.5)	1.00 (0.78–1.28)	1.000
Skåne	33.1 (32.1–34.1)	33.3 (32.3–34.3)	30.4 (29.0–31.8)	30.7 (29.3–32.0)	35.6 (34.1–37.1)	35.8 (34.4–37.3)	1.17 (1.10–1.25)	< 0.001
Halland	23.0 (21.3–24.7)	22.4 (20.7–24.1)	23.9 (21.3–26.4)	23.3 (20.9–25.8)	22.2 (19.8–24.5)	21.6 (19.3–23.8)	0.93 (0.80–1.08)	0.360
Västra götaland	17.3 (16.7–18.0)	17.4 (16.8–18.1)	15.5 (14.6–16.3)	15.6 (14.7–16.5)	19.1 (18.1–20.0)	19.2 (18.2–20.1)	1.23 (1.14–1.33)	< 0.001
Värmland	8.9 (7.8–10.0)	8.6 (7.5–9.6)	7.6 (6.2–9.1)	7.4 (6.0–8.9)	10.2 (8.5–11.9)	9.7 (8.1–11.3)	1.33 (1.03–1.73)	0.030
Örebro	11.8 (10.5–13.1)	11.7 (10.5–13.0)	8.6 (7.1–10.2)	8.5 (7.0–10.1)	14.9 (12.9–16.9)	14.8 (12.8–16.8)	1.73 (1.37–2.18)	< 0.001
Västmanland	16.3 (14.8–17.9)	16.1 (14.5–17.6)	18.2 (15.9–20.6)	17.9 (15.6–20.3)	14.5 (12.5–16.6)	14.3 (12.2–16.3)	0.80 (0.65–0.97)	0.022
Dalarna	10.8 (9.5–12.0)	10.3 (9.1–11.4)	9.3 (7.7–10.9)	9.1 (7.5–10.6)	12.2 (10.3–14.0)	11.4 (9.7–13.1)	1.30 (1.03–1.65)	0.024
Gävleborg	19.8 (18.1–21.4)	19.1 (17.5–20.7)	15.3 (13.2–17.4)	14.9 (12.9–17.0)	24.2 (21.6–26.7)	23.2 (20.7–25.6)	1.58 (1.33–1.88)	< 0.001
Västernorrland	15.9 (14.3–17.5)	15.2 (13.7–16.7)	14.4 (12.2–16.5)	13.9 (11.9–16.0)	17.4 (15.1–19.8)	16.5 (14.3–18.7)	1.21 (0.99–1.49)	0.059
Jämtland	20.3 (17.8–22.8)	19.6 (17.2–22.0)	20.7 (17.1–24.2)	20.0 (16.5–23.4)	19.9 (16.4–23.4)	19.2 (15.8–22.6)	0.96 (0.75–1.24)	0.756
Västerbotten	8.3 (7.2–9.4)	8.3 (7.2–9.4)	9.3 (7.6–11.0)	9.3 (7.7–11.0)	7.3 (5.8–8.8)	7.3 (5.8–8.7)	0.79 (0.59–1.04)	0.089
Norrbotten	14.9 (13.4–16.4)	14.4 (13.0–15.9)	15.3 (13.1–17.5)	14.7 (12.6–16.8)	14.6 (12.4–16.7)	14.1 (12.1–16.2)	0.95 (0.77–1.17)	0.642

^a^ Adjusted for age and sex.

^b^ Poisson test.

## 4 Discussion

In this study, we have shown that the incidence of AAFD referred to specialist care in Sweden is high and that there are age‐related and sex‐related differences. We also found substantial regional variations in the incidence. The findings are important considering that AAFD referred to specialist care could be associated with symptoms and disability that may require complex surgical treatment.

High age and high BMI have been linked to AAFD in several previous studies and an increased overall foot load has been suggested as a contributing factor in the development of AAFD. Considering that obesity has become more prevalent [10], the population is aging, and the field of foot and ankle surgery has developed with increased recognition and development of surgical interventions to treat AAFD, we hypothesized that the incidence of referred AAFD had increased.

The higher incidence of AAFD in women is well established, but the factors behind this sex difference are not known. Differences in activity level, shoe wearing habits, and hormonal composition have been proposed to contribute to higher incidence in women [11, 12, 13]. Further research is needed to understand why women, particularly postmenopausal women, are predominantly affected. In future studies, hormonal profiles in affected women could be studied to elucidate potential hormonal influences on the development of AAFD.

Higher age is also a factor with a well‐established correlation to AAFD, probably because of the time tendon degeneration, inflammation, and progressive malformation takes to develop [1, 3, 5, 6, 7, 11, 14, 15, 16, 17, 18, 19]. The relatively high incidence in younger age groups was surprising, but we have previously published data based on the Swedish national quality register for foot and ankle surgery, Swefoot, that confirms that patients as young as 16 years old are not only diagnosed with AAFD but also treated surgically for this condition [5].

Defining characteristic features of patients with a specific disorder is a critical aspect of clinical practice, as such information helps guide clinicians in forming differential diagnoses. Estimating gender‐specific and region‐specific incidence rates provides a foundation for developing and implementing targeted preventive measures and healthcare resource allocation and improving patient outcomes.

We found large regional variations in age‐adjusted and sex‐adjusted incidence rates. One explanation could be regional variations regarding underlying factors known to be associated with AAFD. Differences in the prevalence of obesity and diabetes could be factors contributing to these regional variations. The region Gotland, with the highest AAFD incidence, exhibited a concurrent high prevalence of overweight and diabetes [20, 21]. However, the region Värmland had a low AAFD incidence but an even higher prevalence of overweight and diabetes compared to Gotland. The differences in AAFD incidence do not seem to follow regional trends regarding obesity and diabetes but the way these factors interact needs further study [20, 21]. Another explanation could be regional differences in referral patterns from primary to specialist care. Because the healthcare system is very similar across the regions, it is unlikely that differences in referral thresholds is a major factor. However, differences in proximity to healthcare facilities and local awareness of the condition may contribute to variations in care access. Greater distances to primary care providers can hinder patients' ability to seek timely help, and even when the condition is accurately diagnosed, referrals to specialist care may involve additional travel, which can be both costly and time‐consuming. This could lead some patients, who would otherwise benefit from specialist care, to forgo referrals due to financial or time constraints. Educational disparities may also influence referral patterns, as previous research has highlighted an underutilization of healthcare resources among individuals with lower socioeconomic status [22]. This may result in lower referral rates in regions where patients predominantly belong to lower socioeconomic groups. To explore this further, future studies could stratify data at the municipal level, as socioeconomic variation within regions is often substantial.

During the last decades, there has been an increasing interest in studying genetic components in disease development [1]. Inherited genetic risk factors, such as molecular changes in extracellular matrix and matrix metalloproteinase polymorphisms, have been suggested to affect the risk of posterior tibial tendon dysfunction and subsequently AAFD [23]. It is also impossible to determine whether an adult with a diagnosis of AAFD had a congenital flatfoot deformity that was not diagnosed until adulthood and genetic factors might contribute even further to disease development in these cases. Genetic composition could have some degree of regional variations but the extent to which it may influence incidence of AAFD is not known.

To our knowledge, this is the first nation‐wide study investigating the incidence of referred AAFD. Reporting to the NPR is mandatory for all inpatient and outpatient healthcare facilities in Sweden since 1987 and 2001, respectively, and we believe the data represent close to complete coverage.

Our study has limitations. We only analyzed data about the registered diagnosis code, age, gender, comorbidities, consultation dates, and the healthcare facility where the diagnosis was registered. We are not able to ascertain how the diagnosis was made or whether the basis for diagnosis was clinical or radiographic. Furthermore, the severity of the deformity remains unknown.

In register‐based epidemiological studies, the ICD‐10‐SE classification system is an important tool to help estimate incidences. The reliability of the diagnosis registrations could be high for certain conditions such as noncomplex fractures. However, when used in the context of AAFD, the classification system has its limitations. Patients referred for AAFD may have received a diagnostic code other than M214, for example, M21 (other acquired deformities of limbs), M216 (other acquired deformities of the ankle and foot), and Q665 (congenital pes planus), which would underestimate the incidence rates. Similarly, physician may use the code M214 for conditions that are not true AAFD. There is no diagnosis code for the functional flatfoot, a nonsymptomatic foot, where the arch is visible when the person is not bearing weight and reduced while standing due to overpronation. This nonpathological foot condition should not be referred to specialist care, but they could potentially be referred due to pain in another area and wrongly receive the diagnosis of AAFD.

Most patients who are referred to orthopedic specialists for foot and ankle problems consult doctors subspecialized in this area, but this is not always the case and nuances in coding of foot and ankle conditions are easily overlooked by practitioners with limited exposure to these conditions. If a less experienced practitioner makes the diagnosis, patients with functional flatfoot or not previously diagnosed congenital flatfoot could potentially be wrongly registered as M214. This would lead to overestimation of the incidence rates. However, we do not believe that this type of misclassification constitutes a significant part of the registrations in this study. The large regional variations found in this study may partly be a result of inconsistent diagnostic coding. This highlights the future need to refine the diagnostic codes used for AAFD.

The terminology associated with AAFD also presents challenges, resulting in varying interpretations even among specialists. These factors highlight the importance of standardized terminology and clear guidelines to ensure accurate diagnosis and treatment of AAFD. Confusing terminology has been suggested as a factor contributing to the difficulty of estimating the prevalence of AAFD [1]. The term posterior tibial tendon dysfunction and its staging system has sometimes been used synonymously with AAFD. The first classification system was developed in 1989 by Johnson and Strom [24]. Several researchers later refined the classification system and began to use the term posterior tibial tendon dysfunction interchangeably with AAFD [6, 12, 25, 26, 27]. In 2020, Myerson et al. published a consensus statement introducing a new nomenclature and classification system aiming to resolve this issue and proposed the term progressive collapsing foot deformity. The authors described progressive collapsing foot deformity as a more complex three‐dimensional deformity involving multiple structures of the foot. Further, since the posterior tibial tendon itself does not seem to be the sole explanation for the deformity, the term posterior tibial tendon dysfunction was removed from the classification system [28]. Since the data analyzed in this study was collected before 2020, we used the term AAFD. However, we chose to exclude the ICD‐10 code used for posterior tibial tendon dysfunction, M768, since it is also used for other enthesopathies of the lower limb, excluding the foot, which made it impossible to determine whether the registrations of this code referred to posterior tibial tendon dysfunction.

In this study, we described AAFD referred to specialist care in Sweden. Patients managed in primary care were not included, as they are not registered in the NPR. To obtain a more accurate estimation of the true incidence of AAFD in the general population, future studies could also explore regional quality registers for primary care to estimate the incidence in that setting. However, considering the challenges associated with diagnosing and accurately registering AAFD in specialist care, these difficulties are likely to be even more pronounced for primary care physicians, potentially affecting the reliability of the data.

The incidence of AAFD on a national level has, to our knowledge, not been reported before. Information about incidence and prevalence of the disease is important to prioritize healthcare resources. This information can be used in future research investigating the possible underlying factors behind the regional variations and the possible role of environmental factors, sex, and comorbidities in disease etiology.

## 5 Conclusion

The incidence of adult acquired flatfoot deformity referred to specialist care in Sweden is high. The incidence rate is approximately two times higher in women compared to men and the highest incidence is seen in the age Group 61–75 years. There are large regional variations in the incidence rate, which needs further investigation.

## Author Contributions

**Ida Osbeck:** conceptualization, data curation, investigation, methodology, project administration, visualization, writing–original draft preparation, writing–review and editing. **Maria Cöster:** conceptualization, methodology, project administration, supervision, writing–review and editing. **Isam Atroshi:** conceptualization, investigation, methodology, project administration, supervision, visualization, writing–review and editing.

## Conflicts of Interest

The authors declare no conflicts of interest.

## Data Availability

Data subject to third party restrictions. The data that support the findings of this study are available from the National Patient Register. Restrictions apply to the availability of these data, which were used under license for this study. Deidentified data are available from the corresponding author upon reasonable request.
